# Concizumab prophylaxis in persons with hemophilia A or B with inhibitors: patient-reported outcome results from the phase 3 explorer7 study

**DOI:** 10.1016/j.rpth.2024.102476

**Published:** 2024-06-17

**Authors:** Huyen Tran, Sylvia von Mackensen, Aby Abraham, Giancarlo Castaman, Kingsley Hampton, Paul Knoebl, Silvia Linari, Jan Odgaard-Jensen, Jesper Skov Neergaard, Oleksandra Stasyshyn, Jay Jay Thaung Zaw, Bulent Zulfikar, Amy Shapiro

**Affiliations:** 1 Ronald Sawers Haemophilia Centre, The Alfred Hospital, Melbourne, Victoria, Australia; 2 Australian Centre for Blood Diseases, Monash University, Melbourne, Victoria, Australia; 3 Department of Medical Psychology, University Medical Centre Hamburg-Eppendorf, Hamburg, Germany; 4 Department of Hematology, Christian Medical College, Vellore, India; 5 Center for Bleeding Disorders and Coagulation, Department of Oncology, Careggi University Hospital, Florence, Italy; 6 Department of Haematology, Royal Hallamshire Hospital, Sheffield, UK; 7 Division of Hematology and Hemostasis, Department of Medicine 1, Medical University of Vienna, Vienna, Austria; 8 Biostatistics HTA, Novo Nordisk A/S, Søborg, Denmark; 9 Safety Surveillance CVD I, Novo Nordisk A/S, Søborg, Denmark; 10 Institute of Blood Pathology and Transfusion Medicine, Lviv, Ukraine; 11 Medical & Science Rare Bleeding Disorders, Novo Nordisk A/S, Søborg, Denmark; 12 Division of Pediatric Hematology-Oncology, Istanbul University Oncology Institute, Istanbul, Turkey; 13 Indiana Hemophilia and Thrombosis Center, Indianapolis, Indiana, USA

**Keywords:** concizumab, health-related quality of life, hemophilia A, hemophilia B, patient preference, patient-reported outcomes

## Abstract

**Background:**

Patient-reported outcomes (PROs) reflect patient perceptions of disease and treatment and are important for evaluating new therapies.

**Objectives:**

Evaluate the effects of once-daily concizumab prophylaxis on health-related quality of life (HRQoL), treatment burden, and treatment preference in males aged ≥12 years with hemophilia A/B with inhibitors.

**Methods:**

Patients enrolled in the multicenter, open-label explorer7 phase 3 study (ClinicalTrials.gov identifier: NCT04083781) were randomized to receive no prophylaxis (arm 1) or concizumab prophylaxis (arm 2) or were nonrandomly allocated to concizumab prophylaxis (arms 3 and 4). The study included questionnaires to assess patients’ perception of HRQoL (Haemophilia Quality of Life Questionnaire for Adults), treatment burden (Hemophilia Treatment Experience Measure), and treatment preference (Haemophilia Patient Preference Questionnaire).

**Results:**

The estimated treatment difference between patients receiving concizumab prophylaxis vs no prophylaxis at week 24 for Haemophilia Quality of Life Questionnaire for Adults “total score” was −22.6 points (95% CI, −42.5; −2.7), directionally favoring patients receiving concizumab prophylaxis. For Hemophilia Treatment Experience Measure “total score,” the estimated treatment difference was −19.9 points (95% CI, −34.3, −5.6) in favor of concizumab vs no prophylaxis. The majority of patients receiving concizumab expressed a preference for concizumab over their previous treatment, the main reasons being “fewer bleeds,” “require less time,” and “less painful to inject.” Across all PROs, there were less responses collected than anticipated, limiting interpretations.

**Conclusion:**

PROs collected during the explorer7 study showed improvements in some domains of HRQoL, treatment burden, and patient treatment preference in persons with hemophilia A or B with inhibitors receiving concizumab prophylaxis compared with no prophylaxis.

## Essentials

• Concizumab is in development as once-daily, subcutaneous prophylactic treatment for hemophilia.
• Patient-reported outcomes were collected by questionnaires in the phase 3 explorer7 study.
• Health-related quality of life, treatment burden, and treatment preference data are presented.
• Patient-reported outcomes favored concizumab prophylaxis over no prophylaxis in many of the domains analyzed.

## Graphical abstract

**Figure:**
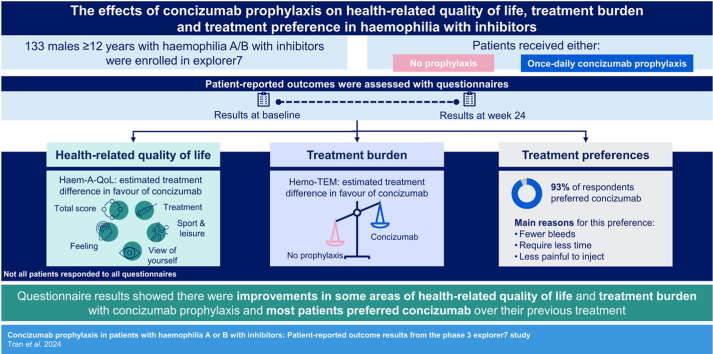

## 1 Introduction

Patient-reported outcomes (PROs) are an important way of evaluating a patient’s health status, with information reported directly by the patient [[1], [2], [3]]. PROs can provide insights on patients’ health-related quality of life (HRQoL) [2,4,5], activity and physical functioning [[6], [7], [8]], or treatment-related adherence, satisfaction, or burden [[9], [10], [11], [12]]. For persons with hemophilia, both the burden of disease as well as the burden of treatment can impact overall HRQoL, particularly in terms of pain and physical functioning [[13], [14], [15], [16]]. In this context, PROs can provide an opportunity to directly assess how persons with hemophilia feel and function and thereby understand how a new treatment with a novel mechanism of action impacts their HRQoL and treatment experience.

Among persons with hemophilia who develop neutralizing antibodies (inhibitors) against factor replacement products, HRQoL may be further reduced due to more frequent bleeding episodes and the associated limitations in everyday activities [14,17,18]. Persons with hemophilia complicated by inhibitors and their caregivers also have a substantial treatment burden [3,10,11]. Prophylaxis with bypassing agents has been used in the past with inconsistent results, especially for persons with hemophilia B (HB) with inhibitors (HBwI) [3,19]. In these patients, there is a need for frequent and regular intravenous administration [3]. Altering the route of treatment administration, for example, to subcutaneous injection, has represented a major advance for persons with hemophilia A (HA) with inhibitors (HAwI) [20]. Reducing treatment burden may in general improve treatment adherence, provide better disease outcomes, and improve the overall HRQoL in persons with hemophilia, particularly those with inhibitors.

Concizumab is a humanized, monoclonal antitissue factor pathway inhibitor (TFPI) antibody in development as a once-daily subcutaneous prophylaxis for patients with HA or HB with or without inhibitors [21]. TFPI acts as an anticoagulant, binding to the tissue factor–activated factor (F)VII complex and inhibiting the initiation phase of coagulation [22]. Concizumab interferes with TFPI binding to the tissue factor–FVIIa complex, increasing activated FX and the formation of prothrombinase. This results in increased thrombin generation to achieve hemostasis despite factor deficiency in patients with HA/HB with or without inhibitors [21,23]. The phase 3 explorer7 study (ClinicalTrials.gov identifier: NCT04083781) evaluated efficacy, safety, pharmacokinetics, and pharmacodynamics, as well as PROs of concizumab prophylaxis in persons with HAwI/HBwI [24].

Results from the explorer7 primary analysis cutoff (when all patients in arm 1 had completed ≥24 weeks of treatment or withdrawn and patients in arm 2 had completed ≥32 weeks of treatment or withdrawn) demonstrated that once-daily subcutaneous concizumab prophylaxis reduced the estimated mean annualized bleeding rate (ABR) compared with no prophylaxis (ABR, 1.7 [95% CI, 1.0; 2.9] vs 11.8 [95% CI, 7.0; 19.9], respectively; ABR ratio, 0.14 [95% CI, 0.07; 0.29]; *P* < .001) in patients with HAwI or HBwI [24]. Adverse events associated with concizumab were mostly of low-grade severity, and serious adverse events were rare [24].

The explorer7 study also evaluated the impact of prophylactic concizumab treatment on patients’ HRQoL. Numerous tools, both generic and disease-specific, are available for measuring HRQoL and treatment experience in persons with hemophilia. The rapidly changing hemophilia treatment landscape means it is important to evaluate treatment efficacy in the context of overall improvements in HRQoL and treatment burden, which requires additional outcome measures beyond bleeding rate and joint examination [25,26]. In the explorer7 study, results from 8 PRO questionnaires (Table) were analyzed as secondary or exploratory endpoints and are reported here.

**Table:** Summary of patient-reported outcome questionnaires used in the phase 3 explorer7 concizumab study.

Endpoints	Questionnaire	Concept	Analyses
Key secondary endpoint	SF-36v2^a^ [4]	Generic health state measure, capturing physical and mental health; 36 items belonging to 8 domains are included: physical functioning, role-physical functioning, bodily pain, general health, vitality, social functioning, role-emotional functioning, and mental health. Two component scores are also calculated, a physical component score and a mental component score. Scores range from 0 to 100, with higher scores indicating better HRQoL.	Changes in SF-36v2 domains “bodily pain” and “physical functioning” from baseline to week 24
Exploratory endpoints	Haem-A-QoL [27,28]	Hemophilia-specific questionnaire that assesses HRQoL over the past 4 weeks in adults (≥17 years); 46 items pertaining to 10 domains are included in this questionnaire, which capture physical, emotional, and social components of HRQoL. A total score is calculated, along with scores for the following domains: physical health, feelings, view of yourself, sport and leisure, work and studies, dealing with hemophilia, treatment, future, family planning^b^ and partnership and sexuality. Scores range from 0 to 100, with lower scores indicating better HRQoL.	Changes in Haem-A-QoL “total score” and “physical health” domain score from baseline to week 24
	PROMIS Short Form v2.0 Upper Extremity 7a [8]	Generic measure of physical functioning in upper limbs; contains 7 questions on physical functioning in the upper extremities, each scored from 1 to 5; higher scores indicate a higher level of physical functioning.	Change from baseline to week 24
	PROMIS Numeric Rating Scale v1.0 Pain Intensity 1a	Generic measure of average pain intensity experienced in the past 7 days from 0 (no pain) to 10 (worst imaginable pain); higher scores indicate higher pain intensity.	Change from baseline to week 24
	Hemo-TEM [10]	Hemophilia-specific tool to assess the burden of hemophilia treatment; 26 items are evaluated in 5 domains: injection difficulties, physical impact, treatment bother, interference with daily life, and emotional impact; scores range from 0 to 100; higher scores indicate a higher treatment burden.	Change in “total score” from baseline to week 24
	H-PPQ^a^	Assesses patient’s hemophilia treatment preference using questions related to preference, including the reasons for, and strength of, their preference.	Patient preference at week 24
	PGI-S on physical functioning	Generic, single-item measure of overall physical functioning. Patients choose the response that best describes their level of physical functioning over the previous 7 days from 1 of 5 options available.	PGI-S score at week 24
	PGI-C in physical functioning	Generic, single-item measure of change in overall physical functioning. Patients choose the response that best describes their overall change in level of physical functioning after starting treatment with the study medication; 7 response options are available.	PGI-C score at week 24

Haem-A-QoL, Haemophilia Quality of Life Questionnaire for Adults; Hemo-TEM, Hemophilia Treatment Experience Measure; H-PPQ, Haemophilia Patient Preference Questionnaire; PGI-C, Patient Global Impression of Change; PGI-S, Patient Global Impression of disease Severity; PROMIS, patient-reported outcome measurement information system; SF-36v2, 36-Item Short Form Health Survey version 2.

a Main findings from SF-36v2 and H-PPQ are reported elsewhere [24].

b This domain was not calculated due to missing items.

## 2 Methods

### 2.1 Study design

The explorer7 study is a prospective, multicenter, open-label phase 3 study evaluating the safety and efficacy of once-daily subcutaneous concizumab prophylaxis in patients with HAwI/HBwI [24]. The study included male patients with HAwI/HBwI aged ≥12 years who had previously been prescribed or needed a bypassing agent in the 24 weeks prior to screening. Patients were randomized into 2 arms (arm 1, no prophylaxis; and arm 2, concizumab prophylaxis) or assigned to nonrandomized arms (arms 3 and 4, concizumab prophylaxis). Patient treatment regimens before entering the study were as follows: on-demand treatment with bypassing agents for arms 1 and 2, prior concizumab treatment during the explorer4 study (ClinicalTrials.gov identifier: NCT03196284) for arm 3, and on-demand treatment or prophylaxis with bypassing agents in arm 4. Patients received a 1.0 mg/kg loading dose of concizumab, followed by an initial daily dose of 0.20 mg/kg. Based on concizumab plasma concentration measured at 4 weeks of exposure to concizumab, the daily dose could be increased to 0.25 mg/kg or decreased to 0.15 mg/kg, if required [24]. Upon completion of at least 24 weeks of participation, patients previously receiving on-demand treatment in arm 1 could receive concizumab prophylaxis.

This study was conducted in accordance with the Declaration of Helsinki, applicable International Council for Harmonisation of Technical Requirements for Pharmaceuticals for Human Use Good Clinical Practice Guidelines, and applicable laws and regulations. Written informed consent was obtained from all patients or their legal representatives prior to any study-related activities. This study was approved by an independent ethics committee or institutional review board as required according to local regulations. An independent Data Monitoring Committee reviewed and evaluated data at predefined timepoints and on an ad hoc basis, and provided recommendations regarding ongoing study conduct to protect patient safety.

### 2.2 Study objectives and endpoints

The primary objective of the explorer7 study was to compare the efficacy of concizumab prophylaxis vs no prophylaxis in reducing the number of bleeding episodes in patients with HAwI/HBwI. Secondary objectives investigated PROs using the 36-Item Short Form Health Survey version 2 (SF-36v2), safety, and pharmacokinetic and pharmacodynamic parameters [24]. Exploratory objectives and endpoints evaluated the impact of concizumab prophylaxis on HRQoL, treatment burden, and treatment preference, as detailed in the Table.

### 2.3 PRO questionnaires

Eight PRO questionnaires, both generic and hemophilia-specific, were included in explorer7 to assess HRQoL, treatment burden, and treatment preference (Table). The hemophilia-specific questionnaire, Haemophilia Quality of Life Questionnaire for Adults (Haem-A-QoL), evaluates HRQoL in the past 4 weeks on a 5-point Likert scale (ranging from “never” to “all the time”) across different domains in adults, with lower scores indicating better HRQoL. The Patient-Reported Outcome Measurement Information System (PROMIS) Numeric Rating Scale v1.0 Pain Intensity 1a questionnaire is a generic measure of the average pain intensity experienced in the past 7 days from 0 (no pain) to 10 (worst imaginable pain). The PROMIS Short Form v2.0 Upper Extremity 7a questionnaire is a generic measure of physical functioning in the upper limbs containing 7 questions with a 5-point scale (“unable to do” to “without any difficulty”); higher scores indicate a higher level of physical functioning. Patient Global Impression of overall disease Severity and impact on physical function (PGI-S) and Patient Global Impression of Change in physical function (PGI-C) are generic, single-item measures to evaluate physical functioning over the previous 7 days using a 5-point scale (“very good” to “very poor”) or the change in the level of physical functioning after starting treatment using a 7-point scale (“very much better” to “very much worse”), respectively. The Hemophilia Treatment Experience Measure (Hemo-TEM) assesses the burden of hemophilia treatment using a 5-point scale (“never” to “always” or “not at all” to “extremely”) over different domains; lower scores indicate lower treatment burden. The Haemophilia Patient Preference Questionnaire (H-PPQ) assesses different aspects of patient treatment-related preference, including the reasons and the strength of their preferences.

### 2.4 PRO questionnaire completion and statistical analysis

Patients completed the PRO questionnaires in their local language, if available, at site visits using an eDiary. Exceptions were Haem-A-QoL (in all countries) and PROMIS (in some countries), which were completed on paper. The endpoints related to Haem-A-QoL, Hemo-TEM, and PROMIS were scored according to their respective scoring algorithms.

The questionnaire completion rates were lower than expected across all PROs. Therefore, the preplanned multiple imputation analysis could not be performed and post hoc statistical methods were applied. The change in statistical model meant that only patients who had completed a questionnaire at both baselines and ≥1 visits postbaseline were included in the statistical analysis.

Haem-A-QoL, PROMIS Numeric Rating Scale v1.0 Pain Intensity 1a, and PROMIS Short Form v2.0 Upper Extremity 7a were completed at baseline and weeks 4, 8, 16, and 24 (Haem-A-QoL only by patients aged ≥17 years). Haem-A-QoL and PROMIS data for patients in arms 1 and 2 were analyzed using a post hoc mixed model for repeated measures (MMRM) to examine changes in scores from baseline to week 24. Treatment (arm 1, no prophylaxis; or arm 2, concizumab prophylaxis), type of hemophilia (HAwI or HBwI), and bleeding frequency (<9 or ≥9 bleeding episodes during the 24 weeks prior to screening) were used as factors, and baseline value as a covariate, all nested within a week (week 4, 8, 16, and 24). An unstructured covariance matrix was used to describe variability for the repeated measurements for a patient. Treatment differences between arms 1 and 2 at week 24 were estimated.

Patients completed the Hemo-TEM questionnaire at baseline and week 24. Answers from respondents were transformed to a score from 0 to 100 within several domains as a measure of treatment burden. An analysis of covariance (ANCOVA) with treatment, type of hemophilia, and bleeding frequency prior to screening were used as factors, and the baseline value as a covariate was applied to evaluate mean change in Hemo-TEM scores from baseline to week 24 for patients in arms 1 and 2. Treatment differences between arms 1 and 2 at week 24 were estimated.

The PGI-S questionnaire was administered at baseline and weeks 4, 8, 16, and 24 and PGI-C at weeks 4, 8, 16, and 24. The H-PPQ was administered at week 24. Patients were asked to select the responses that best reflected their experiences. Data from these questionnaires are presented as number and percentage of patients with responses in each category.

## 3 Results

### 3.1 Study population

The study population of explorer7 has been described previously, including baseline characteristics [24]. A total of 133 patients were enrolled and either randomized or allocated to study arms 1 to 4, and PRO responses were collected from patients during the period from baseline to week 24 (Supplementary Table S1).

### 3.2 HRQoL (Haem-A-QoL)

Statistical analyses to determine the estimated treatment difference (ETD) were based on patients with data at baseline and ≥1 visits postbaseline in arm 1 (*n* = 4) and arm 2 (*n* = 13); in some domains, fewer patients responded (“sport and leisure” [arm 1, *n* = 3; arm 2, *n* = 9] and “work and studies” [arm 1, *n* = 4; arm 2, *n* = 9]). Too few patients contributed data to the “family planning” domain, and therefore, statistical analysis could not be performed on this domain. Although this questionnaire was designed for adults, 1 patient aged <17 years filled out the questionnaire at baseline and postbaseline, and data are included in the statistical analysis.

**Figure 1:**
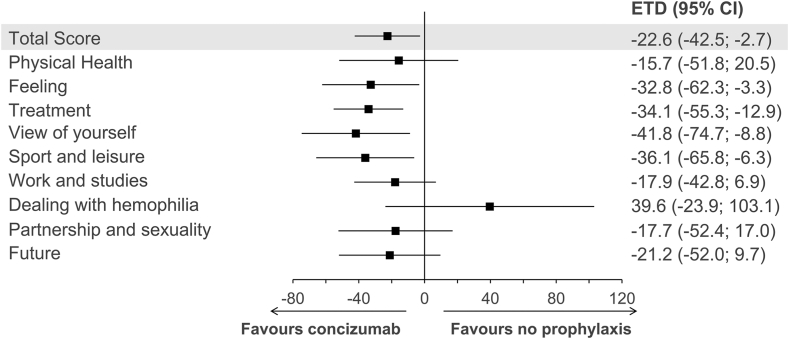
Health-related quality of life measured with Haemophilia Quality of Life Questionnaire for Adults in persons with hemophilia A/B with inhibitors receiving no prophylaxis (arm 1) or concizumab prophylaxis (arm 2). Estimated treatment difference (ETD) in Haemophilia Quality of Life Questionnaire for Adults “total score” (shaded) and domain scores at week 24 in patients receiving concizumab prophylaxis vs no prophylaxis are shown. The ETD was analyzed using a mixed model for repeated measures and based on patients with data at baseline and ≥1 visits postbaseline in arm 1 (*n* = 4) and arm 2 (*n* = 13); in some domains, fewer patients responded (“sport and leisure” [arm 1, *n* = 3; arm 2, *n* = 9] and “work and studies” [arm 1, *n* = 4; arm 2, *n* = 9]). Analysis for the domain “family planning” was not performed due to insufficient data.

In the Haem-A-QoL questionnaire, lower scores correspond to better HRQoL. Mean Haem-A-QoL scores at week 24 were generally unchanged from baseline in arm 1 and lower than baseline in arms 2, 3, and 4 (Supplementary Figure S1). The ETD at week 24 between patients in arm 2 and arm 1 was −22.6 points (95% CI, −42.5; −2.7) for the Haem-A-QoL “total score” and −15.7 points (95% CI, −51.8; 20.5) for “physical health” (Figure 1). The ETD in the domains “feeling,” “treatment,” “view of yourself,” and “sport and leisure” directionally favored concizumab, while the ETD in other domains showed no preference (Figure 1).

### 3.3 Pain and upper extremity functioning (PROMIS)

PROMIS pain intensity and upper extremity functioning results were based on MMRM analysis of data from patients in arm 1 (*n* = 9) and arm 2 (*n* = 22). The mean pain intensity score estimate at week 24 was 3.3 (95% CI, 1.9; 4.7) and 2.4 (95% CI, 1.5; 3.2) for arm 1 and arm 2, respectively, with a mean change from baseline of −0.9 points (95% CI, −2.3; 0.5) in arm 1 and −1.8 points (95% CI, −2.6; −0.9) in arm 2 (Supplementary Table S2). The mean upper extremity functioning score estimate at week 24 was 36.4 (95% CI, 31.4; 41.3) and 42.0 (95% CI, 38.9; 45.1) in arm 1 and arm 2, respectively, with a mean change from baseline of −3.2 points (95% CI, −8.1; 1.8) in arm 1 and 2.5 points (95% CI, −0.6; 5.6) in arm 2 (Supplementary Table S3).

### 3.4 PGI-S and PGI-C

For PGI-S, patients who had completed the questionnaire at both baseline and week 24 were evaluated for their change in responses over time. Among those who responded, 6/7 (86%) patients in arm 1 reported no change in their level of physical functioning from baseline to week 24. Of those who responded, 12/19 (63%) patients in arm 2 and 33/58 (57%) patients in arms 2 to 4 reported better physical functioning (≥1 category improvement) at week 24 than at baseline (Supplementary Table S4).

Among those who responded to the PGI-C questionnaire, 1/11 (9%) patients in arm 1 reported “moderately better,” and 10/11 (91%) reported “no change” in their level of physical functioning at week 24 (Supplementary Table S5). In arm 2, 14/23 (61%) patients reported “very much better,” 6/23 (26%) “moderately better,” 2/23 (9%) “a little better,” and 1/23 (4%) “no change” in levels of physical functioning at week 24. There was a similar pattern of results observed for patients in arms 2 to 4 (*n* = 83).

### 3.5 Treatment burden (Hemo-TEM)

**Figure 2:**
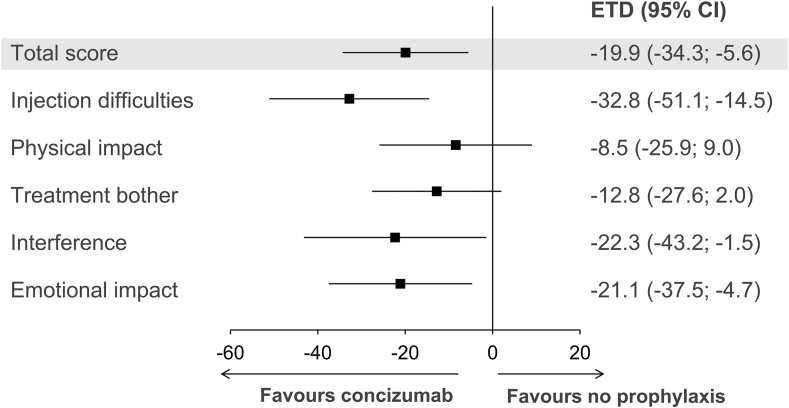
Patient-reported treatment burden measured with Hemophilia Treatment Experience Measure in persons with hemophilia A/B with inhibitors receiving no prophylaxis (arm 1) or concizumab prophylaxis (arm 2). Estimated treatment difference (ETD) in Hemophilia Treatment Experience Measure “total score” (shaded) and domain scores at week 24 in patients receiving concizumab prophylaxis vs no prophylaxis are shown. The ETD was analyzed using analysis of covariance and based on patients with responses at baseline and ≥1 visits postbaseline in arm 1 (*n* = 6) and arm 2 (*n* = 19).

In the Hemo-TEM questionnaire, lower scores correspond to lower treatment burden. Mean Hemo-TEM scores at week 24 were generally unchanged or higher than baseline in arm 1 and lower than baseline in arms 2, 3, and 4 (Supplementary Figure S2). Hemo-TEM statistical analysis was based on patients with data at baseline and ≥1 visits postbaseline (arm 1, *n* = 6; arm 2, *n* = 19). The ETD at week 24 between patients in arm 1 and arm 2 was −19.9 points (95% CI, −34.3; −5.6) for “total score.” For the remaining domain scores analyzed, the ETD in the domains “injection difficulties,” “interference,” and “emotional impact” favored concizumab, while the ETD in other domains (“physical impact” and “treatment bother”) showed no preference (Figure 2).

### 3.6 Treatment preference (H-PPQ)

**Figure 3:**
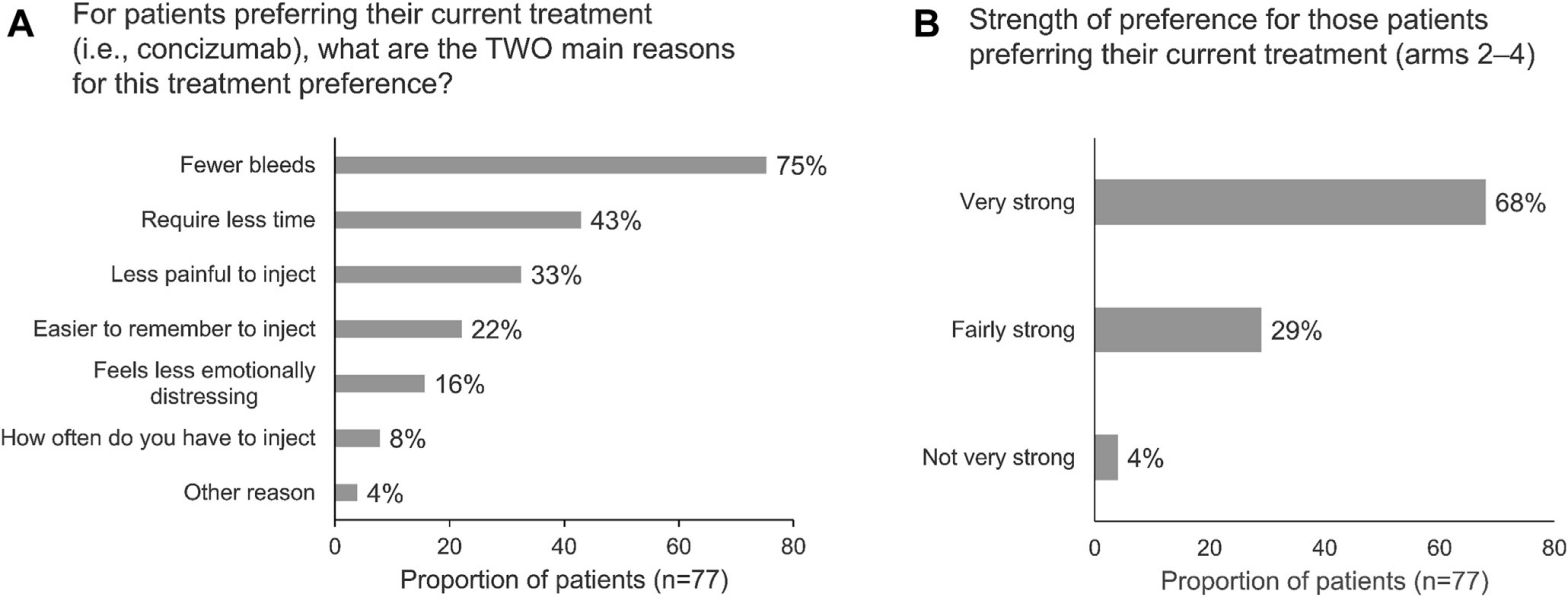
Patient-reported treatment preferences evaluated with Haemophilia Patient Preference Questionnaire in persons with hemophilia A/B with inhibitors receiving concizumab prophylaxis (arms 2-4) who responded to the questionnaire (*n* = 83 from a total of 99 patients). Patients who preferred their current treatment (ie, concizumab, *n* = 77) were asked to provide the 2 main reasons for their preference (A) and the strength of their preference (B).

Patient treatment preference was analyzed using the H-PPQ. As previously reported, from a total of 99 patients in arms 2 to 4, 16 (16%) did not respond to the questionnaire [24]. Of the respondents, 77/83 (93%) preferred concizumab, 5/83 (6%) reported no preference, and 1/83 (1%) preferred their previous treatment [24]. The strength of treatment preference for those favoring their current treatment (ie, concizumab) was reported as “very strong” by 52/77 (68%) patients, “fairly strong” by 22/77 (29%) patients, and “not very strong” by 3/77 (4%) patients (Figure 3A). When asked to select the 2 main reasons for their treatment preference, the most common reasons patients selected for preferring concizumab were “fewer bleeds” (75%), “require less time” (43%), and “less painful to inject” (33%; Figure 3B). The patient who preferred his previous treatment reported this as a “very strong” preference for the reasons that it “feels less emotionally distressing” and “less painful to inject.”

## 4 Discussion

The results from the explorer7 study support the efficacy and safety of concizumab as a once-daily prophylactic treatment for patients with HAwI/HBwI [24]. The exploratory patient-reported endpoint results provided herein give information from the patients’ perspective of the potential benefits of daily subcutaneous concizumab prophylaxis, compared with no prophylaxis, in the context of HRQoL and treatment experience.

### 4.1 Effects of concizumab on patient HRQoL

HRQoL can be analyzed using generic and hemophilia-specific questionnaires. In the explorer7 study, both the hemophilia-specific Haem-A-QoL and generic SF-36v2 HRQoL questionnaires were used. Patients receiving concizumab prophylaxis reported improved HRQoL after 24 weeks compared with those receiving no prophylaxis as determined by an ETD of −22.6 points in the Haem-A-QoL “total score.” The previously published key secondary endpoints, change in SF-36v2 “bodily pain” and “physical functioning” from baseline to week 24, were not significantly different between arms 1 and 2. The ETD of other domains within the SF-36v2 were directionally in favor of concizumab prophylaxis vs no prophylaxis (eg, general health, vitality, role-emotional, mental health, and the mental health component score) [24].

In line with these results, when evaluating HRQoL, pain intensity, and upper extremity functioning, the ETD at week 24 directionally favored concizumab prophylaxis over no prophylaxis in both Haem-A-QoL and PROMIS questionnaires. In addition, improvements in the global assessments of physical function were reported by most respondents receiving concizumab prophylaxis, whereas the majority of respondents on no prophylaxis indicated no change in their overall impression of physical function after 24 weeks of treatment. However, as these results were only based on the patients who had responded to the PGI questionnaires, they are not broadly generalizable and may be subject to responder bias.

### 4.2 Effects of concizumab on patients’ treatment burden

Results from the Hemo-TEM questionnaire indicated that patients receiving concizumab prophylaxis experienced a reduction in their treatment burden. These results align with the earlier explorer4 and explorer5 (ClinicalTrials.gov identifier: NCT03196297) studies investigating concizumab prophylaxis in patients with HAwI/HBwI and severe HA, respectively, which also demonstrated a reduction in treatment burden for patients receiving concizumab prophylaxis [29].

Treatment administration has a substantial physical and emotional impact on patients [10,30]. Key aspects of the treatment burden include injection difficulties, such as venous access issues, interference with daily life due to frequent injections, and the time required for treatment preparation and administration [10]. Issues contributing to treatment burden may include both the route and mode of administration. In contrast to current factor replacement therapies and bypassing agents that are administered by intravenous injection, concizumab is administered subcutaneously using a prefilled, multidose, pen-injector device. The less invasive subcutaneous route has potential advantages over intravenous administration, including easier administration, reduced pain, and reduced time of administration, and may, therefore, improve treatment adherence and patient quality of life by reducing treatment burden [30]. Emicizumab, a bispecific antibody bridging activated FIX and FX to restore hemostasis, is approved for prophylactic treatment of HA and HAwI and is administered via subcutaneous injection once every 2 or 4 weeks [31]. Emicizumab has been reported to reduce the treatment burden for patients with HA, as well as improve aspects of HRQoL [32]. Together with the PROs collected for patients receiving concizumab in the present study, these results suggest that the subcutaneous route of administration may improve the treatment experience of persons with hemophilia.

### 4.3 Patient treatment preference

The observed impact of concizumab on both disease and treatment burden is also reflected in the results from the patient treatment preference questionnaire. The majority of patients receiving concizumab prophylaxis in explorer7 expressed a preference for this treatment compared with their previous treatment [24]. This preference appeared to be driven by both the efficacy and the delivery of the treatment (subcutaneous administration with a pen-injector device) based on patient responses that “fewer bleeds,” “requires less time,” and are “less painful to inject” were the main reasons for their preference. It is interesting that patients reported that concizumab prophylaxis required less time than their previous treatment, even with daily administration. A previous study regarding the pen-injector reported an overall positive response from persons with hemophilia, including ease of use, with fewer steps than their current prophylactic treatment [33]. A separate usability study evaluated the concizumab pen-injector experience in persons with hemophilia and caregivers and showed that using the pen-injector could be learned quickly (<10 minutes on average), preparation and injection time was fast (<2 minutes on average), and most patients found the pen-injector “easy” or “very easy” to use [34]. These characteristics may contribute to patients’ perception that treatment administration “requires less time” than their previous treatment. In addition, pain from injections is generally reduced by using a short, thin needle [35]. In the present study, concizumab was administered with a 4-mm, 32 G needle, which may have contributed to the response that it was “less painful to inject.”

### 4.4 Limitations

The explorer7 study was open-label, and as patients were aware of their treatment allocation, this may be a source of potential bias in the PRO results. In addition, selection bias by study investigators could potentially have impacted the PRO results, as patients in arms 3 and 4 were not randomized. In terms of the results obtained from patients in arms 3 and 4, which also showed a benefit of concizumab prophylaxis on some areas of HRQoL and treatment burden, it should be noted that patients were not randomized, and those in arm 3 had previously participated in the explorer4 study, potentially making these groups not directly comparable with arm 2.

One factor that may have introduced bias to the data was the new baseline that was established after the explorer7 study was paused [24]. In March 2020, concizumab treatment in explorer7 and explorer8 (ClinicalTrials.gov identifier: NCT04082429) was paused due to the occurrence of thromboembolic events [24]. In arms 2 to 4, patients stopped concizumab treatment during the pause and resumed treatment once the study restarted. However, this was not the case for patients in arm 1 who continued to receive on-demand treatment. For the patients in arms 2 to 4 who restarted, “previous treatment” was defined as the treatment they received during the pause. Therefore, these patients had a new baseline after previously having been introduced to concizumab.

Missing data are another factor that may have contributed to bias. The amount of missing data reduced the statistical power and limited our ability to make inferences based on the data collected. The main reasons for missing data included a lack of visit confirmation in the study management system and patients not completing the practice diary at their initial visit, resulting in baseline values being lost. It should also be noted that while it is important to understand a new treatment regimen from a patient’s perspective [3], the collection of such information can also place an additional burden on patients. In this study, patients were asked to complete 8 questionnaires, and questionnaire burden may have contributed to the missing data [36,37]. Similar problems were found in other studies, such as the Expanding Communications on Hemophilia A Outcomes registry, which had to be closed after 2 years due to objections of investigators and patients to the burden of multiple PROs, among other challenges [38].

The analyses for PROs were not performed on an intention-to-treat population. Due to the number of patients with missing data, post hoc statistical methods replaced the originally planned multiple imputation analyses with an MMRM, which meant that only patients with data available at baseline and ≥1 visits postbaseline were included for the comparison of arm 1 vs arm 2. The analysis assumed that all patients who did not complete the questionnaires would have experienced the same benefit as those who did complete the questionnaires, with similar baseline characteristics (treatment arm, type of hemophilia, and bleeding frequency [<9 or ≥9 bleeding episodes during the 24 weeks prior to screening]).

Lastly, the timeframe of 24 weeks used in this study for assessing improvements in pain and physical functioning may have been too short to capture some changes, particularly joint-related improvements.

## 5 Conclusions

Despite the questionnaire completion rate being lower than anticipated, the PRO data collected in this study directionally favored concizumab prophylaxis vs no prophylaxis in some areas of HRQoL. Reductions in treatment burden and patient treatment preference favored concizumab prophylaxis over no prophylaxis. The PRO results included here support the potential value of once-daily subcutaneous concizumab prophylaxis as a treatment option for persons with HAwI/HBwI.
