# Supplementary material for: Concizumab prophylaxis in persons with hemophilia A or B with inhibitors: patient-reported outcome results from the phase 3 explorer7 study
Source: Res Pract Thromb Haemost. 2024 Jun 17;8(4):102476. doi: 10.1016/j.rpth.2024.102476 (PMC11295565; doi:10.1016/j.rpth.2024.102476)
Supplement: Supplementary material [file mmc1.docx]

**Supplementary information**

**Title:**

Concizumab prophylaxis in patients with haemophilia A or B with inhibitors: Patient-reported outcome results from the phase 3 explorer7 study

**Authors:**

Huyen Tran*^,†,a^, Sylvia von Mackensen ^‡^_,_ Aby Abraham^§^, Giancarlo Castaman^¶^, Kingsley Hampton**, Paul Knoebl^††^, Silvia Linari^¶^, Jan Odgaard-Jensen^‡‡^, Jesper Skov Neergaard^‡‡^, Oleksandra Stasyshyn^§§^, Jay Jay Thaung Zaw^‡‡^, Bulent Zulfikar^¶¶^, Amy Shapiro***

**Affiliations:**

*Ronald Sawyers Haemophilia Centre, The Alfred Hospital, Melbourne, Australia;

^†^Australian Centre for Blood Diseases, Monash University, Melbourne, Australia;

^‡^Department of Medical Psychology, University Medical Centre Hamburg-Eppendorf, Hamburg, Germany;

^§^Department of Hematology, Christian Medical College, Vellore, India;

^¶^Center for Bleeding Disorders and Coagulation, Department of Oncology, Careggi University Hospital, Florence, Italy;

**Department of Haematology, Royal Hallamshire Hospital, Sheffield, UK;

^††^Division of Hematology and Hemostasis, Department of Medicine 1, Medical University of Vienna, Vienna, Austria;

^‡‡^Novo Nordisk A/S, Søborg, Denmark;

^§§^Institute of Blood Pathology and Transfusion Medicine, Lviv, Ukraine;

^¶¶^Division of Pediatric Hematology-Oncology, Istanbul University Oncology Institute, Istanbul, Turkey;

***Indiana Hemophilia & Thrombosis Center, Indianapolis, IN, USA.

**Supplementary material:**

- Supplementary methods
- 5 supplementary tables
- 2 supplementary figures

*Additional mitigation measures after the treatment pause*

The key mitigation measure implemented was guidance for the treatment of mild and moderate breakthrough bleeds regarding use of the lowest approved dose for additional haemostatic agents. Patients were required to contact the study site before treatment of a suspected bleed. For severe or life-threatening bleeding episodes, specific recommendations were not considered feasible, as management of these bleeds presents complex clinical challenges that may need to be addressed on a case-by-case basis, which may require frequent, high dosing of bypassing agents for extended periods of time. In the rare event of a severe/life threatening bleed, the patient was to be in immediate and close contact with the study investigator and be treated with relevant doses of factor-containing products at the discretion of the study investigator.

## Supplementary Table S1: Patient-reported outcome questionnaires and number of respondents in the explorer7 study

|  | **Randomised (n)** | | | | **Non-randomised (n)** | | | |
| --- | --- | --- | --- | --- | --- | --- | --- | --- |
|  | **Arm 1 (no prophylxis)** | | **Arm 2 (concizumab prophylxis)** | | **Arm 3^e^ (concizumab prophylxis)** | | **Arm 4^f^ (concizumab prophylxis)** | |
| FAS | 19 | | 33 | | 21 | | 60 | |
| FAS and ADS | 19 | | 29 | | 15 | | 55 | |
| Completed 24/32 weeks of treatment | 14 | | 28 | | 15 | | 53 | |
| Completed at PACO | 13^c^ | | 27^d^ | | 15 | | 53 | |
|  | **Baseline** | **Week 24** | **Baseline** | **Week 24** | **Baseline** | **Week 24** | **Baseline** | **Week 24** |
| **SF-36v2** | 10 | 12 | 24 | 24 | 13 | 14 | 34 | 52 |
| **Haem-A-QoL^a^** | 8 | 4 | 14 | 10 | 15 | 9 | 28 | 21 |
| **Hemo-TEM** | 9 | 11 | 23 | 23 | 12 | 14 | 31 | 51 |
| **H-PPQ^b^** | NA | NA | NA | 23 | NA | 14 | NA | 46 |
| **PGI-S** | 10 | 12 | 23 | 23 | 12 | 14 | 27 | 46 |
| **PGI-C** | NA | 11 | NA | 23 | NA | 14 | NA | 46 |
| **PROMIS-Pain** | 10 | 12 | 23 | 23 | 13 | 14 | 32 | 51 |
| **PROMIS-UE** | 11 | 12 | 24 | 22 | 12 | 12 | 21 | 41 |

133 patients with HAwI or HBwI were enrolled in explorer7. Patients in arm 1 completed 24 weeks and patients in arms 2–4 completed 32 weeks. The n used for statistical analyses varied by questionnaire.

^a^Only for patients ≥17 years of age; fewer patients completed questions in the domains ‘sport and leisure’ in (arm 1: n=8 at baseline, n=3 at Week 24; arm 2: n=11 at baseline, n=7 at Week 24; arm 3: n=15 at baseline; n=8 at Week 24; arm 4: n=21 at baseline; n=18 at Week 24); ‘work and studies’ in (arm 2: n=11 at baseline, n=7 at Week 24; arm 3: n=12 at baseline, n=7 at Week 24; arm 4: n=23 at baseline; n=17 at Week 24), ‘view of yourself’ (arm 4: n=27 at baseline; n=21 at Week 24) and ‘treatment’ (arm 4: n=27 at baseline; n=20 at Week 24). Analysis for the domain ‘family planning’ was not performed due to insufficient data.

^b^H-PPQ was only administered at Week 24 to patients receiving concizumab in arms 2–4.
^c^One patient withdrew after completing 24 weeks.

^d^One patient died after restart and after completing 32 weeks (Covid-19).

^e^Patients transferred from explorer4 to arm 3.

^f^Patients on prophylaxis or on-demand treatment with bypassing agents were screened following completion of recruitment to arms 1 and 2 and were assigned to arm 4.

ADS, analysis data set; FAS, full analysis set; Haem-A-QoL, Haemophilia Quality of Life Questionnaire for Adults; haemophilia HAwI, haemophilia A with inhibitors; Hemo-TEM, Hemophilia Treatment Experience Measure; HBwI, haemophilia B with inhibitors; H-PPQ, Haemophilia Patient Preference Questionnaire; NA, not administered; PACO, primary analysis cut off (when all patients in arm 1 had completed at least 24 weeks and all patients in arm 2 had completed at least 32 weeks); PGI-C, Patient Global Impression of Change; PGI-S, Patient Global Impression of disease Severity; PRO, patient-reported outcome; PROMIS-Pain, patient-reported outcome measurement information system numeric rating scale v1.0 pain intensity 1a; PROMIS-UE, PROMIS short form v2.0 upper extremity 7a; SF-36v2, 36‑Item Short Form Health Survey version-2

## Supplementary Table S2: PROMIS Numeric Rating scale v1.0 - Pain Intensity 1a questionnaire responses from patients receiving no prophylaxis (arm 1) or concizumab prophylaxis (arm 2)

|  | **Arm** **1 (no prophylaxis)** | **Arm** **2 (concizumab prophylaxis)** |
| --- | --- | --- |
| **N contributing to analysis** | 9 | 22 |
| Mean score estimates at Week 24 (95% CI) | 3.3 (1.9; 4.7) | 2.4 (1.5; 3.2) |
| Mean change from baseline score estimates at Week 24 (95% CI) | −0.9 (−2.3; 0.5) | −1.8 (−2.6; −0.9) |
| Difference estimate at Week 24  (95% CI) | −0.9 (−2.6; 0.7) | |

Average pain intensity experienced in the past seven days from 0 (no pain) to 10 (worst imaginable pain) was estimated using a mixed model for repeated measures (MMRM) for patients with results at baseline and >1 visits post-baseline.

CI, confidence interval; PROMIS, patient-reported outcome measurement information system

## Supplementary Table S3: PROMIS Short Form v2.0 Upper Extremity 7a questionnaire responses from patients receiving no prophylaxis (arm 1) or concizumab prophylaxis (arm 2)

|  | **Arm 1  (no prophylaxis)** | **Arm 2  (concizumab prophylaxis)** |
| --- | --- | --- |
| **N contributing to analysis** | 9 | 22 |
| Mean score estimates at Week 24 (95% CI) | 36.4 (31.4; 41.3) | 42.0 (38.9; 45.1) |
| Mean change from baseline score estimates at Week 24 (95% CI) | −3.2 (−8.1; 1.8) | 2.5 (−0.6; 5.6) |
| Difference estimate at Week 24  (95% CI) | 5.7 (−0.2; 11.5) | |

Higher scores in the PROMIS Short Form v2.0 Upper Extremity 7a indicate a higher level of physical functioning, which was estimated using a mixed model for repeated measures (MMRM) for patients with results at baseline and >1 visits post-baseline.

CI, confidence interval; PROMIS, patient-reported outcome measurement information system

## Supplementary Table S4: Analysis of changes in the PGI‑S questionnaire responses from baseline to Week 24

|  | **No prophylaxis** | **Concizumab prophylaxis** | | | |
| --- | --- | --- | --- | --- | --- |
|  | **Arm 1 n (%)** | **Arm 2 n (%)** | **Arm 3 n (%)** | **Arm 4 n (%)** | **Arms 2–4 n (%)** |
| **Total patients, n** | 19 | 29 | 15 | 55 | 99 |
| Patients not included due to missing data*, n (%) | 12 (63) | 10 (34) | 3 (20) | 28 (51) | 41 (41) |
| **Patients with baseline and Week 24 data, n (%)** | 7 (100) | 19 (100) | 12 (100) | 27 (100) | 58 (100) |
| 2-category worsening | 0 | 0 | 0 | 1 (4) | 1 (2) |
| 1-category worsening | 0 | 0 | 2 (17) | 4 (15) | 6 (10) |
| No change | 6 (86) | 7 (37) | 5 (42) | 6 (22) | 18 (31) |
| 1-category improvement | 1 (14) | 9 (47) | 4 (33) | 8 (30) | 21 (36) |
| ≥2-category improvement | 0 | 3 (16) | 1 (8) | 8 (30) | 12 (21) |
| **≥1-category improvement^†^** | **1 (14)** | **12 (63)** | **5 (42)** | **16 (59)** | **33 (57)** |

Analysis is based on the the number of patients with data at both baseline and Week 24, and catergorised based on the number of categories improved. *Patients with missing data are noted as a percentage of the total patients in each arm, excluding any data collected before the treatment pause. ^†^The number of patients with ≥1-category improvement summarises all patients reporting improvements in PGI‑S from baseline to Week 24. Patients were allocated to the non-randomised arms 3 and 4, and results may not be directly comparable.
n, number of respondents; %, percentage of respondents

## Supplementary Table S5: Patient global impression of change (PGI‑C) in physical functioning since baseline

|  | **No prophylaxis** | **Concizumab prophylaxis** | | | |
| --- | --- | --- | --- | --- | --- |
|  | **Arm 1 n (%)** | **Arm 2 n (%)** | **Arm 3 n (%)** | **Arm 4 n (%)** | **Arms 2–4 n (%)** |
| **Total patients, n** | 19 | 29 | 15 | 55 | 99 |
| Patients not included due to missing data*, n (%) | 8 (50) | 6 (21) | 1 (7) | 9 (16) | 16 (16) |
| **Number of patients included, n (%)** | 11 (100) | 23 (100) | 14 (100) | 46 (100) | 83 (100) |
| **Response** |  |  |  |  |  |
| Very much better | 0 | 14 (61) | 5 (36) | 27 (59) | 46 (55) |
| Moderately better | 1 (9) | 6 (26) | 5 (36) | 13 (28) | 24 (29) |
| A little better | 0 | 2 (9) | 4 (29) | 2 (4) | 8 (10) |
| No change | 10 (91) | 1 (4) | 0 | 4 (9) | 5 (6) |
| A little worse | 0 | 0 | 0 | 0 | 0 |
| Moderately worse | 0 | 0 | 0 | 0 | 0 |
| Very much worse | 0 | 0 | 0 | 0 | 0 |

Patients were asked to rate the ‘Overall change in your level of physical functioning since you started taking the study medication’ using one of the options listed in the table. Results from Week 24 are presented. *Patients with missing data are noted as a percentage of the total patients in each arm, excluding any data collected before the treatment pause. Patients were allocated to the non-randomised arms 3 and 4, and results may not be directly comparable.
n, number of respondents; %, percentage of the total number of respondents

## Supplementary Figure S1: Haem‑A‑QoL mean scores at baseline and Week 24


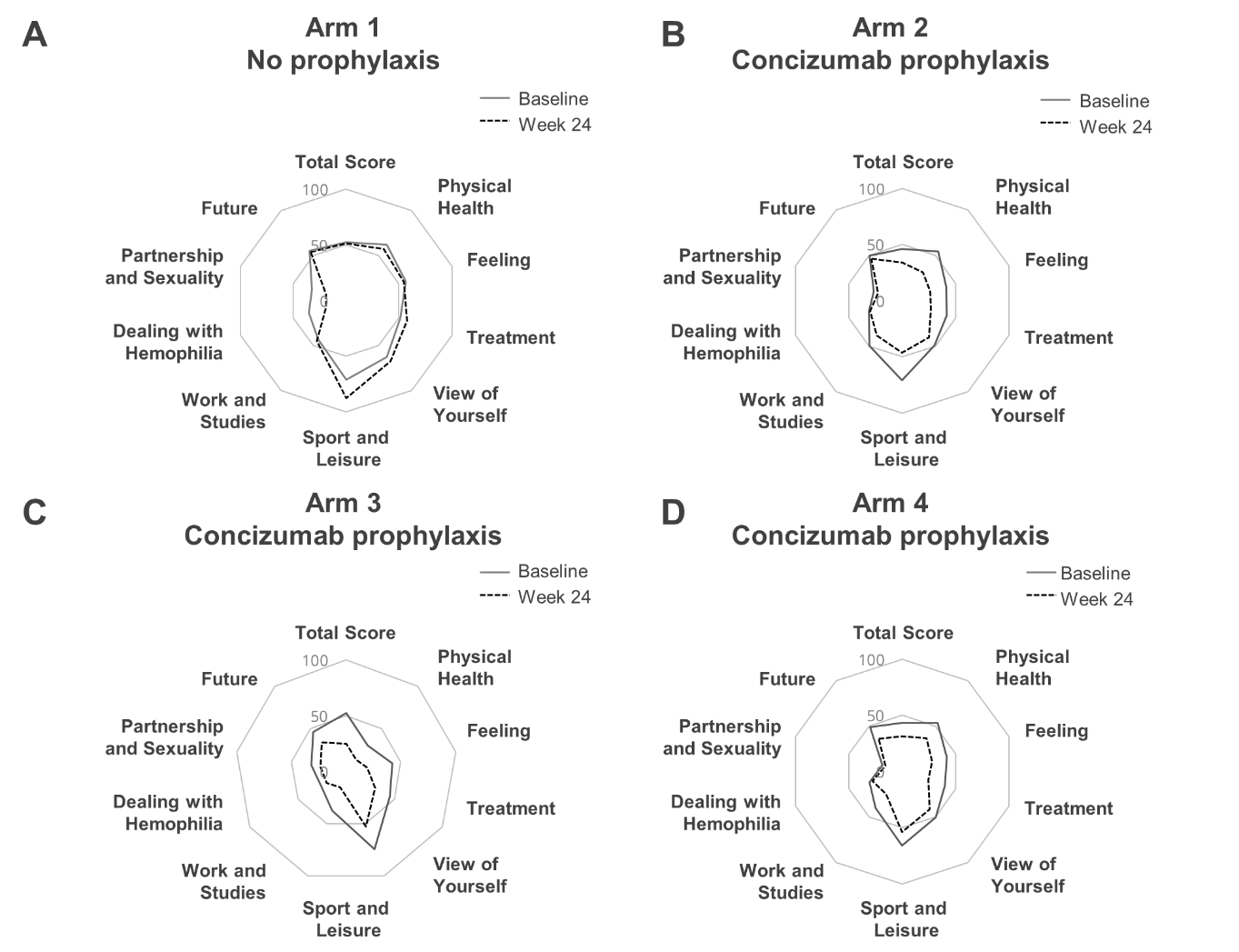


Radar plots show the mean Haem‑A‑QoL scores for the different domains at baseline and at Week 24 for patients in the randomised arm 1 (A; n=8 at baseline, n=4 at Week 24) and arm 2 (B; n=14 at baseline, n=10 at Week 24), and non-randomised arm 3 (C; n=15 at baseline, n=9 at Week 24) and arm 4 (D; n=28 at baseline, n=21 at Week 24). Lower scores indicate better HRQoL (corresponding to smaller radars). Domains with less patient responses: ‘view of yourself’ (arm 4, n=27 at baseline; n=21 at Week 24); ‘sport and leisure’ (arm 1, n=8 at baseline; n=3 at Week 24; arm 2, n=11 at baseline; n=7 at Week 24; arm 3, n=15 at baseline; n=8 at Week 24 and arm 4, n=21 at baseline; n=18 at Week 24); ‘work and studies’ (arm 2, n=11 at baseline; n=7 at Week 24; arm 3, n=12 at baseline; n=7 at Week 24 and arm 4, n=23 at baseline; n=17 at Week 24); ‘treatment’ (arm 4, n=27 at baseline; n=20 at Week 24). Patients were allocated to the non-randomised arms 3 and 4, and results may not be directly comparable. Patients who received prior concizumab treatment during the explorer4 study were allocated to arm 3; patients who received on‑demand treatment or prophylaxis with bypassing agents were allocated to arm 4.
Haem‑A‑QoL, Haemophilia Quality of Life Questionnaire for Adults; HRQoL, health-related quality of life

## Supplementary Figure S2: Hemo‑TEM mean scores at baseline and Week 24


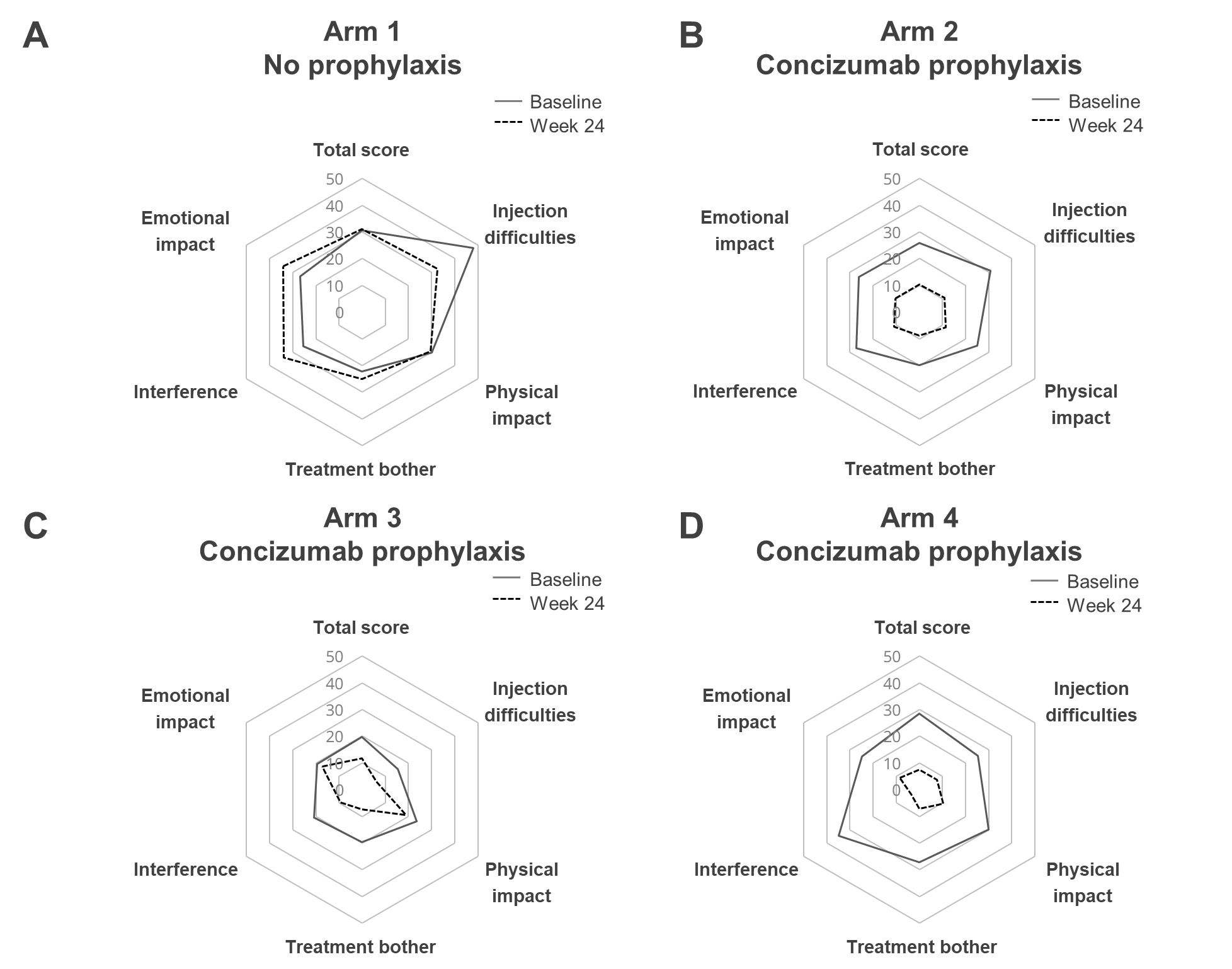


Radar plots show mean Hemo‑TEM domain scores at baseline and at Week 24 for patients in the randomised arm 1 (A; n=9 at baseline; n=11 at Week 24) and arm 2 (B; n=23 at baseline and Week 24); and non-randomised arm 3 (C; n=12 at baseline n=14 at Week 24) and arm 4 (D; n=31 at baseline, n=51 at Week 24). Lower scores indicate lower treatment burden (corresponding to smaller radars). Patients were allocated to the non-randomised arms 3 and 4, and results may not be directly comparable. Patients who received prior concizumab treatment during the explorer4 study were allocated to arm 3; patients who received on‑demand treatment or prophylaxis with bypassing agents were allocated to arm 4.
Hemo‑TEM, Hemophilia Treatment Experience Measure
